# Influence of Hemp Shives Size on Hygro-Thermal and Mechanical Properties of a Hemp-Lime Composite

**DOI:** 10.3390/ma13235383

**Published:** 2020-11-27

**Authors:** Przemysław Brzyski, Mateusz Gładecki, Monika Rumińska, Karol Pietrak, Michał Kubiś, Piotr Łapka

**Affiliations:** 1 Faculty of Civil Engineering and Architecture, Lublin University of Technology, 40 Nadbystrzycka St., 20-618 Lublin, Poland; mateusz.gladecki@pollub.edu.pl (M.G.); monika.ruminska@pollub.edu.pl (M.R.); 2 Institute of Heat Engineering, Faculty of Power and Aeronautical Engineering, Warsaw University of Technology, 21/25 Nowowiejska St., 00-665 Warsaw, Poland; karol.pietrak@pw.edu.pl (K.P.); michal.kubis@pw.edu.pl (M.K.); piotr.lapka@pw.edu.pl (P.Ł.)

**Keywords:** hemp-lime, shives, thermal conductivity, vapor permeability, water absorption, mechanical properties

## Abstract

Different fractions of hemp shives are used in the mixtures of the hemp–lime composite. The market offers shives of different granulation. It depends on the type of industrial hemp and on the capabilities of decortication machines. The aim of the research presented in the article is to check differences in the mechanical and hygro-thermal properties of composites with different shives fractions. The research part of the paper presents the preparation method and investigation on hemp–lime composites. Apparent density, total porosity, thermal conductivity, capillary uptake, vapor permeability, specific heat, mass absorptivity, flexural and compressive strength were examined. The results confirm that the shives fraction influences the individual properties of the composites. Hemp–lime composites with fine shives are characterized by higher water absorption, thermal conductivity, mechanical strength, vapor permeability as well as lower capillary-lifting capacity and specific heat than composites with thick shives.

## 1. Introduction

The hemp–lime composite is an insulating material based on a lime binder and a filler in the form of pieces of the wooden part of the industrial hemp stem called hemp shives. Hemp lime composite is usually used as an insulating material for filling a timber frame wall. By choosing the right mixture it is also possible to use composite as a load-bearing material in the form of wall blocks. Properties of the composite are influenced by many factors, including the proportion of binder to shives, type of binder, and degree of compaction. By reducing the binder content in the mixture, the thermal conductivity [1,2] of the composite and its compressive strength [1] decrease. The type of binder primarily affects mechanical properties [3,4] and setting process [5]. With the increase in compaction force, the thermal conductivity [6] and strength parameters [7] of the composite increase. Hemp shives have also a significant influence on the mechanical and hygro-thermal properties of the composite.

Different fractions of hemp shives are used in the mixtures of the hemp–lime composite. Many countries offer the sale of hemp shives of various fractions and qualities. It depends on the type of industrial hemp and on the capabilities of decortication machines. Dimensions of hemp shives and its level of fragmentation affect the properties of the final composite.

Increasing the content of fine fractions of shives in the composite leads to an increase in the bulk density of the composite [1,8,9]. Stevulova et al. [9,10] used shives with a fraction of 0.063 mm and up to 8 mm and found that compressive strength of composites increases with decreasing mean particle length of hemp shives and that the water absorptivity increases with decreasing mean particle size of hemp shives slices. Arnaud and Gourlay [11] used three shives mixes of the following medium lengths: 3.1, 7.6 and 8.9 mm. They observed that the use of finer shives results in lighter hemp–lime composites whose mechanical properties evolve more slowly due to reduction of macropores size but, finally, have higher compressive strength and Young’s modulus. Bourdot et al. [8] examined composites based on hemp shives in two length ranges: 0–5 mm and 0–20 mm. Shorter shives had the bulk density of 135 kg/m^3^ and the porosity of 89.3%, while longer ones have the density of 110 kg/m^3^ and the porosity of 91.3%. They concluded that the composite with the shives composition of 0–5 mm 30% and 0–20 mm 70%, can be considered as the optimal composition with regards to the mechanical and hygro-thermal properties. Nguyen et al. [7] used two types of shive: completely fibreless shives and those mixed with fibres. Fibered shives present a lower bulk density and a lower water absorption coefficient than pure shives. The authors proved that the composite containing a mixture of shives and fibers is characterized by worse mechanical properties than the composite based on pure shives.

Long fractions of hemp shives (>30 mm) can cause difficulties in mixing and arranging the mix. It is then necessary to thicken and mix the mixture more thoroughly to eliminate technological voids created by randomly arranged longer shives limiting the access of smaller fractions which can fill the space between them [12]. The stronger compaction will lead to an increase in the weight of the composite and a reduction in thermal insulation properties [13].

The use of by-product fine particles, from the hemp industry, as an aggregate into a render could be an efficient way to improve the acoustic and thermal properties [14] of the composite.

In the case of using shives in building composites, their appropriate quality is important. The shives mixture should be thoroughly cleaned of dust. Hemp dusty fractions and dust originating from the ground, due to the larger specific surface area compared to the recommended shives fractions, increase the demand for water and binder during the preparation of the composite mix. The consequence of using a larger amount of binder is, therefore, an increase in the value of the composite thermal conductivity [1,15]. There is a risk that the dusty fractions absorb water that is needed for the crystallization process of the lime particles or the hydration process of the hydraulic components of the binder. In the case of high dust content, the amount of water in the mix should be increased, which will significantly increase the drying time of, e.g., a wall made of composite. Dust from the ground, being contamination, will adversely affect the bonding quality of the binder. The shives should also be cleaned of other foreign contaminants, such as other plants.

The hemp fiber content of the mix is also important. A small amount of fiber may have a positive effect on the characteristics of the composite, acting as a natural micro-reinforcement, while too much of it may lead to an increase in water demand, expansion of the composite and prolonged drying time of the hemp–lime composite partitions. The most undesirable are the fiber clumps formed in the process of mechanical processing of hemp stalks. They pose a threat due to the inability to protect such elements with lime binder. Clumps during mixing are surface covered with lime binder (the binder does not get to the inside of the fiber clumps), while water gets inside, which, stored by unprotected fibers inside the clumps, can cause corrosion and, consequently, degradation of the hemp–lime composite.

The properties of the shives are also influenced by the details of the processing of hemp stems, namely whether the retting process was used or not [16]. The main effect of the retting process is a decrease of the bulk density which is the result of hemp degradation, enhancing its thermal and acoustic performance. The retting process also has a positive effect on the shives’ insulation properties.

Fine particles (<5 mm) will in turn lead to an increased demand for water, but also for the binder due to the larger specific surface area. The consequence will be an increase in density [9,10]. Shives of short lengths also result in weaker bonding of the composite and greater brittleness. However, such fractions are suitable for making plasters based on hemp shives. The plaster uses a greater amount of binder as compared to wall composites. In plasters, finely chopped fibers are also recommended in order to reduce shrinkage cracks caused, e.g., by the drying of the material.

There are several publications, mentioned above, on the influence of the size of the hemp shives on material properties. However, this knowledge is not complete because these research works concern only selected fractions of shives of a specific origin and only selected properties of composites. Therefore, further research is needed to analyze the influence of shive size on the other properties, such as vapor permeability, capillary rise and specific heat of the composite. These effects were analyzed in the current study. In addition to the aforementioned properties, the influence of shives on the basic properties of the composite, such as flexural and compressive strength, water absorption and thermal conductivity, was also checked. The article concerns shives with a different fraction and origin than those presented in the literature.

The target application of the composite proposed in the article is to fill and insulate timber frame walls in a monolithic form or in the form of a prefabricated element (insulation boards). The aforementioned properties that have been examined and analyzed in the article are important for this type of wall material. The main aim of the article is to assess the differences in the properties of the composite made according to two mix designs that differ in the size of the shives used. The results presented in this paper may be helpful in selecting the appropriate fraction of shives for a specific application.

## 2. Materials and Methods

### 2.1. Mix Design

Hydrated lime of CL-90s class (Lhoist, Tarnów, Poland), manufactured in accordance with the PN-EN 459-1 [17], was used as a binder (80% of the total weight of binder mix). It is the most commonly used binder for hemp–lime composites. It is characterized by a high pH value, which is beneficial in combination with an organic filler, as it prevents the development of biological corrosion. This lime is the binder in the slow carbonation process. In order to accelerate the early setting and improve the parameters of the composite, such as mechanical strength and resistance to water, an additive in the form of a reactive pozzolana—metakaolinite (Astra Polska, Gdańsk, Poland) was used (20% of the total weight of binder mix). In this case, the binding will take place while the mixture is placed in the formwork. The binding of the hydrated lime alone would not take place because the formwork panels cut off access to air. The binding would only take place after removing the plates. This material has been investigated before [3,12]. The hemp shives are a highly absorbent filler. In order to ensure the right amount of water for the chemical binding of lime and pozzolana, an admixture ensuring water retention, methylcellulose, was used (0.5% of the total weight of binder mix). This admixture has already been tested in the same application in other studies [3,13]. It has been proven that the use of this admixture improves the parameters of the composite due to a more complete setting process [3].

**Figure 1:**
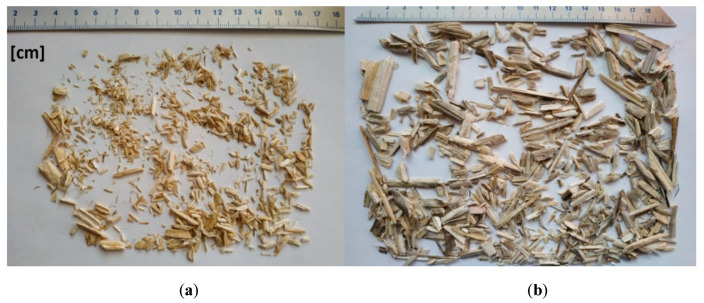
Hemp shives used in the investigation: fine shives (**a**) and thick shives (**b**).

Two types of hemp shive different in fraction and origin were used as shown in Figure 1. The first type (fine) was produced by the Dutch company Hempflax, while the second one (thick) was produced by the Polish company Podlaskie Konopie. The mixtures made of them will be referred to as fine hemp shives (FHS) and thick hemp shives (THS). The shives constituted 50% of the total weight of each binder mix.

The content of shives of individual lengths in the mixture of fine and thick shives is shown in Table 1. The size of the shives was tested on samples weighing 10 g for the thick fraction and 5 g for the fine fraction. They were obtained from material packages delivered by the manufacturers. The measurement was carried out using an electronic caliper and consisted of measuring over 1000 shives fragments from both mixes encompassing the full size spectrum of hemp shives. The mass fractions of constituents in two investigated mix designs are shown in Table 2.

### 2.2. Sample Preparation

The hemp lime mixture was made as follows: the hemp shives were poured into a vessel and some water was added. In turn the dry blended binder mix (hydrated lime and metakaolinite) was inserted. All components were mixed. The remainder of the water mixed with the methylcellulose was then added. The components were mixed until a homogeneous mixture was obtained. The amount of water was determined experimentally, after obtaining the appropriate consistency of the mix. This means that all shives should be wrapped with the moistened binder and the mixture should show a tendency to stick together when forming the “test ball” with the hand. Then the mixture was placed in molds and compacted by hand with wooden rammers.

### 2.3. Density and Total Porosity

In order to characterize the composite, its basic physical features have been determined. The apparent density, specific density and total porosity of the composites were measured. Five samples with dimensions of 100 mm × 100 mm × 100 mm prepared from each mixture were used to test the apparent density. Specific density was determined with the helium gas pycnometer. In that case, larger samples were ground to the form of fine, homogenous powder, then dried at 60 °C for several days. One sample of each type of hemp–lime composite was prepared for the test. Samples had a mass of approx. 20 g. The measurements were conducted according to the PN-EN 12390-7 [18] standard. The total porosity was estimated as a ratio of total volume of open and closed pores per sample volume.

### 2.4. Water Absorptivity

The water absorptivity is the ability to absorb water at atmospheric pressure. Mass absorptivity tests were conducted according to the PN-EN 13755:2008 [19] standard on five 100 mm × 100 mm × 100 mm specimens for each mixture. As there are no standards for a hemp–lime composite or similar material based on plant aggregate, the test was based on the aforementioned standard. However, the time intervals in which the weight gain was read and sample dimensions were selected independently, in order to adapt it to the hemp–lime composites characteristic. The samples were completely submerged in water. Then, the water absorptivity was found as the ratio of the mass of the absorbed water and mass of the dry sample. Volume water absorptivity was also calculated, which is expressed as the volume of water absorbed relative to the sample volume.

### 2.5. Capillary Uptake

This experimental test was conducted in accordance to the PN-EN 1925 standard [20]. As in the case of the water absorption test, the test methodology described in this standard was used, while the dimensions of the samples and the time intervals for reading the weight gain were determined individually. This standard, after adjusting it for the purposes of testing the hemp–lime composite, was also used in other studies [13]. Three samples of each mixture, with the dimensions of 80 mm × 80 mm × 240 mm were placed in contact with water at a depth of approximately 10 mm. In the determined intervals of time, the increase in the mass of samples was recorded. In this way, the amount of water absorbed was determined. An increase of the samples mass was measured in the following intervals of time: 15 min, 30 min, 1 h, 3 h, 6 h, 12 h, 24 h, 3 d, 5 d and 7 d.

### 2.6. Thermal Conductivity

The thermal conductivity test was performed on three specimens of each mixture with the dimensions of 250 mm × 250 mm × 50 mm. The thermal conductivity study was implemented based on international standard ISO 8302 [21]. The study was carried out in the plate apparatus Laser Comp Fox 314 (TA Instruments, New Castle, DE, USA) by using the heat flow meter method. Before the test, the samples were dried to constant mass in a furnace at 60 °C. During the thermal conductivity test, the temperature set on a hot plate was 25 °C, while the cooling plate was at 0 °C, and the average temperature obtained equaled 12.5 °C. The absolute thermal conductivity measurement accuracy of the plate apparatus FOX 314 was ± 2%. The heat in the test flowed through the sample in a direction perpendicular to the compaction direction.

### 2.7. Vapor Permeability

The water vapor permeability was tested using the cup method, as described in norm ISO 12572:2016 [22] with minor modifications. The measurement sets were prepared by fixing the composite samples (100 mm × 100 mm × 37 mm cuboids with ± 2 mm differences) to glass dishes (approx. 100 mm × 100 mm × 100 mm, of cuboid shape, with 3 mm glass thickness) using construction silicone (Selena S.A., Wroclaw, Poland). To assure one-dimensional vapor flow the sides of the samples were also sealed with the silicone. The sealing was additionally secured with duct tape. Two types of substance were used in the dishes, namely calcium chloride (Ciech Soda Polska S.A., Inowroclaw, Poland) (desiccant) and saturated aqueous solution of potassium nitrate (AGNEX, Bialystok, Poland), to assure the relative humidities of 0% and 94% inside the testing assemblies, respectively. The assemblies were placed in the environmental chamber which maintained conditions of temperature of 23 °C and relative humidity of 50%. In such way, for each of two materials, a single value of water vapor permeability was determined for the lower range of relative humidities (0–50%), and a second value for the upper range (50–94%). For each measurement condition two samples were prepared. Prior to the measurements, the samples were conditioned in the environmental chamber in the measurement conditions (23 °C, 50% relative humidity (RH)), until they reached stable mass, as required by the norm ISO 12572:2016 [22].

In the measurement cycle, the mass of assemblies was determined using analytical balance once every 2 or 3 days until the water vapor flux through the samples was stable. In the calculations, the values of the flux averaged from 3 mass measurements were used instead of 5 advised by the norm, but deviations of the flux were minimal, so the modification is believed to be insignificant to the validity of the results. Under given assumptions, the water vapor permeability was calculated based on relations given in the standard ISO 12572:2016 [21] and the vapor diffusion resistance factor based on the calculated water vapor permeability of still air, i.e., μ = δ_a_/δ, where the permeability of air equal to δ_a_ = 1.98 × 10^−10^ was obtained using the equation given by Huang et al. [23].

### 2.8. Specific Heat

The differential scanning calorimeter DSC 404 F1 (Selb, Germay) manufactured by Netzsch was used to determine specific heats of hemp–lime composites. The small size of crucibles is a characteristic feature of the DSC method. Thus, due to limited volume of measuring crucibles compared to the size of microstructural components of hemp–lime composites, a special sample preparation had to be performed. Each type of composite had to be fragmentated into smaller elements and ground to fine powder with a laboratory hammer mill in order to provide a representative and homogenous sample that can be inserted into a DSC crucible. Samples in the form of powder were dried at a temperature of 60 °C for approximately 72 h until no change in mass was detected.

Three samples of each type of composite were prepared from dry powder and their mass was measured with high-precision analytical balance Radwag MYA/2Y (Radwag, Radom, Poland). The mass of each sample was around 16 mg. Each DSC measurement was performed at the same settings:

- Inner atmosphere: argon 20 ml/min,
- Heating rate: 1 K/min,
- Temperature range: 35–75 °C.

During DSC measurements the impact of the air which filled the pores in the composite was omitted after sample grinding. The mass fraction of air in the composite is very small as compared to the mass fractions of shives and binder. Therefore, neglecting it seems to be fully justified.

### 2.9. Compressive Strength

The compressive strength was determined on 3 cubic samples with the following dimensions 150 mm × 150 mm × 150 mm for each mixture, using a hydraulic press MTS 809 (MTS System Corporation, Eden Prairie, MN, USA). Due to the lack of standards for this material, arbitrary assumptions were made regarding the settings of the hydraulic press. In this study the press head was controlled by the displacements with a value of 5 mm/min. However, various displacements of the compression press head were applied in other studies. For example, de Brujin [24] used the same displacement of the compression press head. Williams et al. [1] in turn, used a head displacement of 3 mm/min. Sassoni et al. [25] similarly, but depending on the type of sample, used a head displacement of 3 mm/min or 5 mm/min.

During testing of the samples, the loads and displacements were measured. The compressive force was directed parallel to the direction of compaction of the sample, replicating the reality, because the mixture in the walls also compacts in the direction of the wall rising.

### 2.10. Flexural Strength

Flexural strength was determined on 3 specimens with the following dimensions: 100 mm × 100 mm × 500 mm for each mixture, using a hydraulic press MTS 809 (MTS System Corporation, Eden Prairie, MN, USA). The samples were then loaded with a centrally placed force (3-point-bending). The spacing of the supports was 300 mm. The press head was controlled by the displacements with a value of 0.5 mm/min. On the other hand, Walker et al. [3] in their research followed the standard PN-EN 196-1 [26], assuming the beam load increment to be equal to 10 N/s. In turn, Sassoni et al. [25] assumed an increase in head displacement equal to 10 mm/min while Williams et al. [1] used a head displacement of 3 mm/min.

## 3. Results and Discussion

### 3.1. Density and Total Porosity

The results of measurements are shown in Table 3. Since both mixtures use the same proportions of binder to shives, the bulk density of composites is comparable. The composite with smaller shives is slightly denser, because the mixture with fine shives was more susceptible to compaction. This resulted in a greater amount of binder in 1 m^3^ of the mixture. The results of measurements performed with helium gas pycnometer showed similar values for both types of sample. The absolute difference in the specific density between two types of samples is 97.2 kg/m^3^, while for total porosity 0.5%.

The tested composites were characterized by total porosity of 82.2% (FHS) and 81.7% (THS). The high porosity resulted from the porous structure of the hemp shives (90%) [27], the porous binder, but also from the way of laying and compacting the mixture due to which voids are formed between the shives. The results obtained, regardless of the size of the shives, were comparable with those reported in the literature. For example, according to the research of Rahim et al. [28], the porosity of lime–hemp composites was comparable to our measurements—the composite with a bulk density of 478 kg/m^3^ showed a total porosity of 76.4%. However, in another study [29], the porosity of 80% was obtained with the composite volumetric density of 304 kg/m^3^. In the investigation conducted by Collet and Pretot [30] for composites in the density range of 258–463 kg/m^3^, the total porosity was in the range of 84.9–72%, respectively.

### 3.2. Water Absorptivity

**Figure 2:**
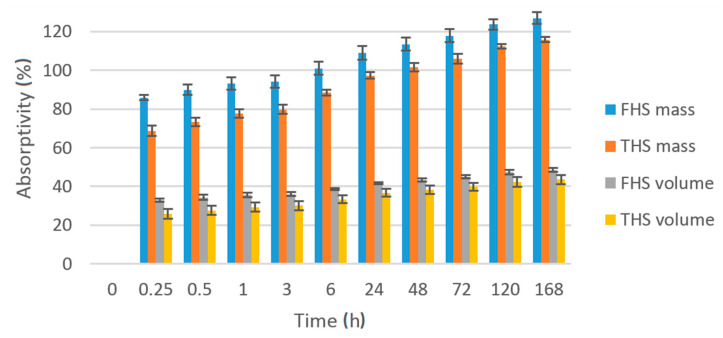
Increase in the volumetric and mass water absorptivity of composite samples over time.

The results of measurements together with the standard deviations are shown in Figure 2. Regardless of the fraction of hemp shives, composites are characterized by high water absorption. The mass water absorption of composites containing fine shives was 126.9% after 1 week of the test, while for composites containing thick shives it was 115.5%. These results are similar to those presented in the literature, e.g., in [12] where composites with a density of 432.6 kg/m^3^ were characterized by mass water absorption of the order of 128%. The biggest differences in the results between the two composites are visible in the initial period of the study. After 15 min of water absorption, the difference was 17.2%, while after 1 week of complete immersion of the samples, the difference dropped to 11.2%. Composites with fine shives are, therefore, characterized by faster water absorption. This may be related to the larger specific surface area of the fillers, but also to their structure. They are crushed into small pieces, which may open the closed pores in the raw material, i.e., the wooden core of the hemp stem. The greatest dynamics of water absorption occurs in the first min after the samples are immersed in water. The composites were immersed in water for 5 min and absorbed 70–80% of the amount of water absorbed overall during the entire test period (7 days). Looking at the results, in walls that are exposed to more intense water, it will be more effective to use thick shives in a hemp–lime mixture. However, the protection of the outer surface of the hemp-lime composite wall is usually made of lime plaster.

High absorptivity of the composites is due to the high total porosity of the shives and their ability to absorb water. The study of the absorbability of hemp shives was carried out by, among others, Arnaud and Gourlay [11]. The shives in a very short time were able to absorb significant amount of water (2–3 times more in relation to the dry mass); 10 min after immersion, their saturation reached 95%. Stevulova et al. [10] studied composites with a much lower water absorption, i.e., 25%, but their density was of the order of 1070 kg/m^3^, shives fraction was 4-8 mm and shives volume composition/binder/water was 40/29/31%.

### 3.3. Capillary Uptake

**Figure 3:**
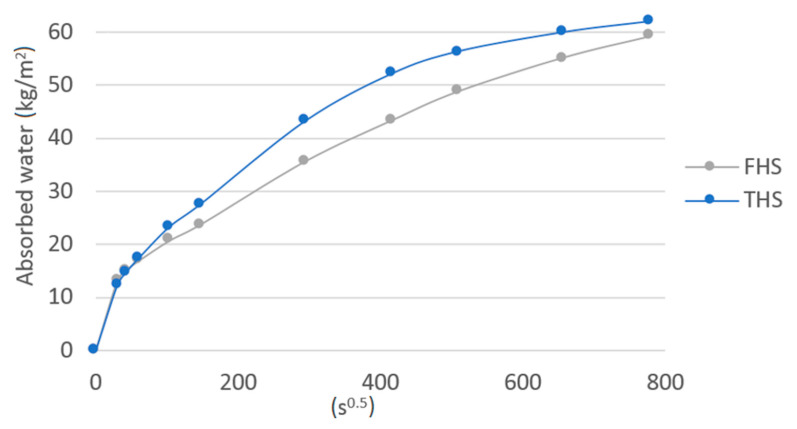
Capillary uptake of tested composites.

The results of capillary uptake are shown in Figure 3. Contrary to the ability to absorb water after immersing the entire sample of the composite, in the case of capillary uptake, the composite containing longer hemp shives was more effective. In the initial period of the study, up to 30 min, the differences between the FHS and THS were imperceptible, while after 1 h, up to 2 days, the differences in the amount of water drawn up increased (THS samples absorbed more water). After two days, the rate of increase in water content in the THS composite decreased, and after 7 days (end of test), the differences in the amount of water absorbed between the two composites were slight. The greater susceptibility to water rising of a composite containing longer shives may be due to larger pores between the shives than in the case of FHS, where shives fractions are smaller and therefore more closely matched to each other. Similar conclusions were drawn in [12], where an additional filler in the form of expanded perlite was used in the composite containing long shives, which filled the spaces between the shives, thus limiting capillary rise. Walker et al. [13], in their research on composites with the same ratio of shives to binder, presented the capillary uptake coefficient after 24 h of the test; it was 2.65 and 3.37 kg/(m^2^h^1/2^) depending on the type of binder. In this study, this coefficient for the 24 h period was higher and equal to 7.27 kg/(m^2^h^1/2^) and 8.82 kg/(m^2^h^1/2^) for the FHS and THS composite, respectively. A similar value of 9 kg/(m^2^h^1/2^) was observed by de Bruijn et al. [24] for higher density concrete samples (from 587 to 733 kg/m^3^). By contrast, other wall-building materials, such as autoclaved aerated concrete with a density of 423 kg/m^3^ had a smaller capillary uptake coefficient equal to 5.28 kg/(m^2^h^1/2^) [31].

### 3.4. Thermal Conductivity

The results of thermal conductivity measurements with the standard deviation are presented in Table 4. Composites containing fine shives are characterized by a higher value of thermal conductivity. This is due to the higher density of these composites compared to the THS. The mixture with fine shives was more susceptible to compaction, the shives were arranged better in form, which made the structure more compact and homogeneous. The result obtained for different FHS samples were similar and therefore a small standard deviation was obtained. In the case of THS composites, the shives were more varied, there were long shives which made it difficult to lay the mixture accurately, making the structure less homogeneous. Finally, a greater discrepancy in the results was obtained. The ability to insulate heat is one of the most important parameters of the hemp composite, and the results obtained prove that one of the proposals for improving this parameter is replacing fine shives with thick shives.

The results obtained are similar to those presented in the literature. For example, Walker and Pavia [13] used the same proportion of binder to shives but the composites were more dense (508–627 kg/m^3^), which resulted in an increase in the thermal conductivity (0.117–0.138 W/(m∙K)). In other studies [1], it was observed that the composite containing shives with an average length of 7.54 mm was characterized by a higher thermal conductivity coefficient than the composite containing shives with an average length of 15.27 mm, although the difference was small and the thermal conductivity value oscillated around 0.12 W/(m∙K).

### 3.5. Vapor Permeability

The results of measurements are presented in Table 5. It may be seen that vapor diffusion resistance factors for the higher relative humidity range are similar to those obtained by Walker and Pavia (5.42 to 5.72) [13], whereas in the lower humidity range the resistance is on average twice as large. To accurately compare these results, it has to be said that in [13] only the lower RH range was measured. Therefore, in fact the currently obtained vapor diffusion resistance factors are greater than those reported by Walker and Pavia [13], even though their materials had greater density (508–627 kg/m^3^).

On the other hand, the current result resembles that reported by Collet et al. [32] both in the range of values and in the fact that the material becomes more permeable in greater humidity. These researchers obtained water vapor permeability of 1.7 × 10^−11^ and 2.3 × 10^−11^ kg/(m·s·Pa) for moulded hemp concrete in lower and higher relative humidity conditions, respectively. In their research, dry condition was kept inside the cup for both cases, and the outside relative humidity was either 50% or 85%. Considering that they used slightly different conditions, the current result may be considered very similar. Their samples possessed density closer to current samples (420 kg/m^3^).

The permeabilities of thick and fine-shiv composites are almost identical in lower RH conditions (see Table 5), and in higher RH conditions, the fine-shiv material is only slightly more permeable. Considering that the result is averaged from a relatively small number of samples (2), the difference may be contained within the range of measurement error. 

Based on studies of the hemp–lime composites with various types of binder (hydrated lime, cement, hydraulic lime, granulated blast furnace slag and metakaolinite), Walker and Pavia [13] concluded that there is no essential difference in vapor diffusion resistance factor for materials with different binders (for all cases µ ≈ 5.5) and that the size of macro-pores between individual hemp shives has greater effect on the vapor permeability than the size of micropores within the hemp shives. By contrast, the authors of earlier studies [33,34] concluded that the type of binder has impact on the water vapor permeability, and the smaller the percentage of the hydraulic material in the binder, the lower its vapor diffusion resistance factor.

### 3.6. Specific Heat

**Figure 4:**
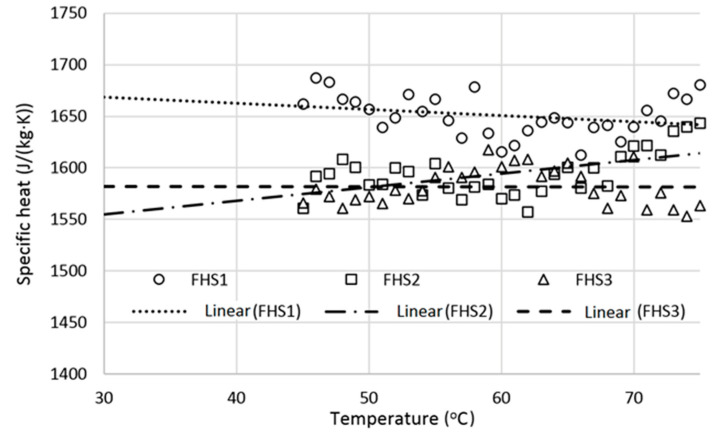
Specific heat vs. temperature for FHS samples.

**Figure 5:**
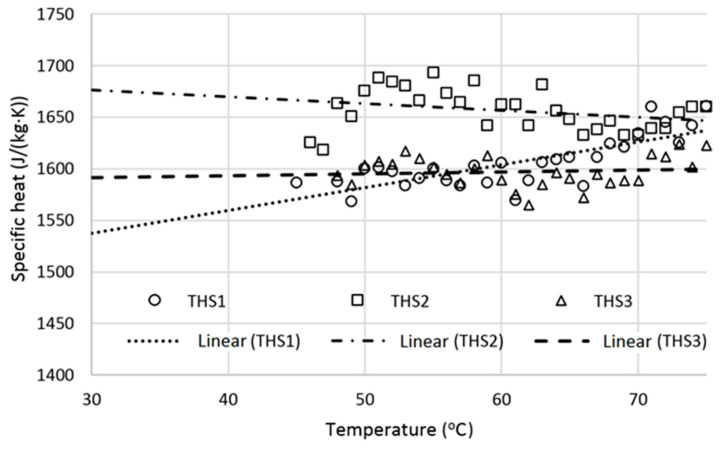
Specific heat vs. temperature for THS samples.

The results of specific heat measurements are shown in Table 6 and in Figure 4 and Figure 5. Specific heats at lower temperatures, down to T = 30 °C, were obtained based on linear extrapolation of the data obtained in the temperature range from 45 to 75 °C (see Figure 4 and Figure 5).

The specific heat of composite materials is sensitive to many factors, e.g., mass ratio of their components or moisture content but usually the spatial arrangement of components is not important if there are no internal stresses. Previous studies have shown that specific heat of hemp-lime composites can be as high as 1300 J/(kg∙K) for materials with apparent density of 508–627 kg/m^3^ [3,29]. Maalaouf et al. [35] reported specific heat of 1100 J/(kg∙K) and sample density equal to 440 kg/m^3^. Values of 1000 J/(kg∙K) and 413 kg/m^3^ may be also found in the literature [36].

It may be seen that the obtained specific heat is higher than the specific heat shown by other authors based on measurements performed with water calorimetry technique. Nevertheless, there is also mentioned a wide range of estimated specific heat from 1100 J/(g∙K) to 1560 J/(g·K) [29] which is similar to results shown in Table 6. 

In general, specific heats of composites with THS and FHS are very similar and vary in the range of 1550 to 1700 J/(kg·K) for the examined temperature range (see Figure 4 and Figure 5). A similar level of specific heats was expected as both composites had the same ratio of shives to binder. The largest difference in specific heat at temperature 30 °C for FHS and THS samples are respectively approximately 12% and 8%. This may indicate slight inhomogeneity due to small samples required for DSC measurement compared to large samples used in water calorimetry technique [3]. However, in the case of the differential scanning calorimeter the sample remains dry during the entire measurement and thus no water adsorption effects occur during the measurement.

### 3.7. Compressive Strength

**Figure 6:**
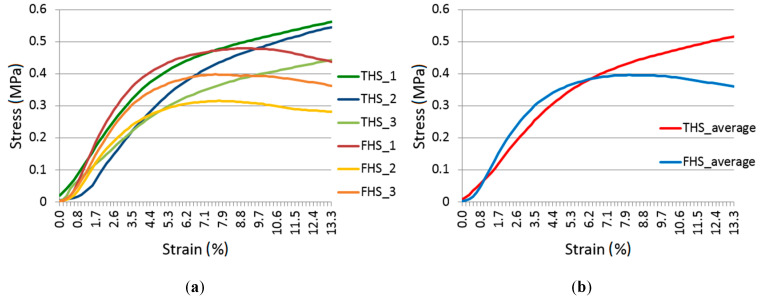
Stress–strain relationship: all samples (**a**) and average values (**b**).

The results of the compressive strength test are shown in Figure 6. The course and trend of the curves for samples from a given mixture are repeatable and differ in the magnitude of the stress at a given strain. However, when comparing the characteristics of the two composites (THS and FHS), it can be noticed that they are different.

Analyzing the characteristics obtained in the compressive strength test of samples from the THS formula, it was found that the destructive stress of the sample cannot be clearly determined, on the basis of which it would be possible to determine the compressive strength of the composite. A similar characteristic as for this mixture was found in the literature [1,37,38]. In Figure 6 it can be seen that in the first phase of loading, the material behaved elastically, the stress increase was significant, while the level of deformation was small. At this stage, the load was mainly taken over by the binder. In the second phase of loading, a change in the characteristic slope can be observed. There is a significant increase in strain with a slow increase in stress. In this phase, after breaking the bond strength, the hemp shives compress, thus eliminating technological pores (air spaces between the shives). The tests were completed with a head displacement of 20 mm (strain equal to 13.3%) because the sample was visually damaged and lost its stability (it could be easily crushed in the hand). The same assumption was made in other studies [37]. However, in our own trial tests it was checked whether, if the study had not been stopped at this point, this phase would have continued even up to 50 mm of displacement. It was verified in the trial tests that in the next phase of loading, when the shives were already compressed, the sample continued to resist the compressive stress and the strain slowly increased.

**Figure 7:**
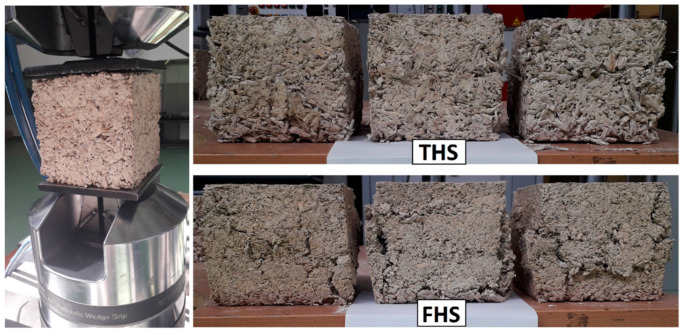
The compressive strength test set-up and the forms of destruction of composite specimens from THS and FHS mixtures.

In the case of FHS samples, the first phase of the characteristic is rectilinear, which proves that the material behaves elastically, and the main role at this point is played by the binder connecting the still uncompressed shives. When the binder fails, the deformation increases with a stabilized value of the compressive force. After reaching the maximum (destructive) stress, the bond strength between the binder on the shives is completely destroyed. The sample then ceases to resist the load and the strain increases noticeably. The compressive strength test set-up and the forms of destruction of composite specimens from THS and FHS mixtures are shown in Figure 7. The behavior of the samples under load shown in Figure 6 is confirmed by the damage patterns of the samples after the compression test shown in Figure 7. The THS samples are compressed, but no excessive damage to the side surfaces is visible, while in the case of FHS samples, the destruction (falling off) of the side surfaces is clearly visible. This failure of the FHS samples shows that the maximum stress has been reached and the samples no longer resist the compressive force. Considering the fact that samples containing fine shives are completely destroyed faster under load, it is more advantageous from a practical point of view to use a thicker fraction of shives.

Stevulova et al. [9] proved that the compressive strength of the hemp-lime composite is higher when it contains fine shives. This conclusion is also reached in other research [1]. It is difficult to confirm this relationship unambiguously in this work, because the maximum force occurred only in the case of FHS composites. If, on the other hand, the stresses at a displacement equal to 5 mm (strain equal to 3.33%) are compared, when the samples of both composites were still elastic, the FHS composite showed higher compressive stress (average value equal to 0.29 MPa) than THS (average value of 0.24 MPa). Similar strengths were obtained in other studies [3,11,24,38,39,40]. The type of shives did not affect the possibility of using the composite as a load bearing material in the walls. The strengths obtained are too low. However, there are several publications presenting the results of compressive strength with higher values. Stevulova et al. [10] proved that it is possible to obtain compressive strengths equal to 1.86 MPa and 2.73 MPa with the use of MgO binder.

### 3.8. Flexural Strength

**Figure 8:**
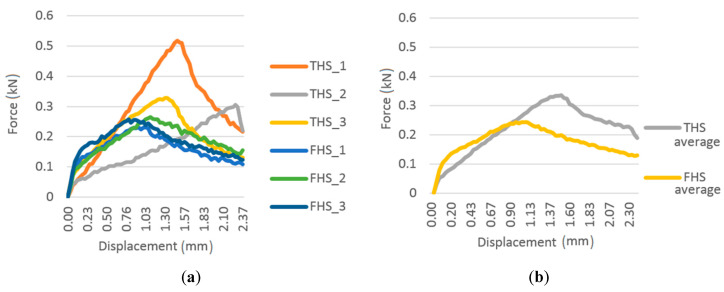
Flexural force-displacement relationship: all samples (**a**) and average values (**b**).

The results of the flexural strength test are shown in Figure 8. In the case of samples from the THS formula, there is a greater variation in the behavior of the samples under static load. One sample was destroyed with a deflection of about 2.30 mm, and the other two with approximately 1.30 mm. In the case of samples from the FHS mixture, the behavior of the samples under increasing load was not very differentiated, which proves a greater homogeneity of the structure of the composite containing fine shives. The failure occurred with a deflection of about 0.8–1.0 mm. The THS samples failed with greater deflection than the FHS samples because the longer shives provided better bonding and worked as dispersed reinforcement. Similar observations are described in [39]. Taking into account the maximum breaking force, the flexural strength of the THS composite was 0.151 MPa, while the FHS composite was 0.110 MPa. However, in the case of the THS composite, the strength of the THS_1 sample differs significantly from the other two, so more samples should be tested to more accurately characterize this composite in terms of the value of the breaking force.

The results obtained are comparable with those obtained in other studies [40] on a lime–hemp composite containing 20% of hemp shives (length 2 and 4 mm) in relation to the total mass of the composite with a volume density of about 600 kg/m^3^. In the cited studies, the results were in the range from 0.08 to 0.141 MPa. In other studies [1], comparing composites with different fractions of shives, composites containing long shives (15.27 mm) were also characterized by higher bending strength than those containing short shives (7.54 mm).

## 4. Conclusions

The paper presents the variation in the hygro-thermal and mechanical properties of hemp–lime composites differing in the fraction of hemp shives, while maintaining the same mass fractions of binder and shives.

A thorough analysis of the results obtained allows is to reach the following conclusions:

- The difference in densities between FHS and THS samples are minor. Samples with fine shives possess a slightly higher density and total porosity than samples with thick shives.
- Regardless of the mixture, the composites are characterized by high mass absorptivity (above 100%). The biggest differences in the results between the two composites are visible in the initial period of the study. Composites with fine shives are characterized by higher and faster absorption of water.
- In the case of capillary uptake, the composite containing longer hemp shives (THS) absorbed more water and the amount of water absorbed after 24 h was higher by about 21% than in the case of FHS.
- Composites containing fine shives are characterized by a higher thermal conductivity (by about 6%) than composites with thick hemp shives.
- Water vapor permeability results of THS and FHS composites were close, which suggests that it is not strongly dependent on shives’ size.
- Both composites possess similar specific heat. Slightly higher values were obtained for the THS composite.
- Both composites behave differently under compressive load. The arrangement of the shives under the influence of compaction determines whether different composite structures can be obtained. In the case of THS it was not possible to establish the maximum destructive force. Comparing the initial behavior of the samples under load (with a displacement of 5 mm), a greater strength, by about 20%, was demonstrated by the composite inflicting incorporating fine shives.
- Flexural strength of the THS composite was higher by about 37%, than the FHS composite. Samples of FHS composites showed greater homogeneity of results and behavior under increasing load compared to THS.

In terms of hygro-thermal properties, both composites behaved similarly. However, FHS composite had a slightly higher value of density due to better compaction ability. This resulted in a slightly higher thermal conductivity and vapor partibility in the relative humidity range of 50–94% for FHS composite than for THS one. On the other hand, specific heat was not affected by shives fraction as it depends on the mass fraction of the constituents which were the same in both materials. In terms of mechanical properties, despite the higher density of the composite containing fine shives, THS composites have better properties. In the case of flexural strength, longer shives (THS mixture) worked more effectively as reinforcement, while in the case of the compression test, the composites from the THS mixture retained greater stability under increasing load.

Taking into account the better thermal parameters, lower mass absorptivity, higher flexural strength and greater stability under compressive load, it can be concluded that it is more effective to use thick shives as a filler in a hemp–lime composite for use as a timber frame wall filling material or insulation prefabricate.

## Figures and Tables

**Table 1:** Sizes of the shives used in the research.

Type of Shives	Fraction (mm)	Average Length (mm)	Average Width (mm)	Max. Length (mm)	Max. Width (mm)
Fine	0–12	2.74	1.42	11.78	4.55
Thick	0–50	8.40	2.68	47.38	11.77

**Table 2:** Mass fractions of constituents in examined mixtures.

Constituents		FHS	THS
Binder	Hydrated lime	80%	
	Metakaolinite	20%	
Filler	Fine hemp shives	100%	-
	Thick hemp shives Filler: binder weight ratio	-	100%
		1:2	1:2
Additive	Methylcellulose	0.5% by weight of the binder	
Water	Water/binder ratio	1.72	1.63

**Table 3:** Averaged values of apparent density, specific density and porosity of samples of hemp–lime composite with the FHS and THS.

Parameter	Unit	FHS	THS
Apparent density	(kg/m^3^)	382.4	376.9
Specific density	(kg/m^3^)	2152.6	2055.4
Total porosity	(%)	82.2	81.7

**Table 4:** Results of thermal conductivity test.

Parameter	Unit	FHS	THS
Thermal conductivity coefficient	(W/(m∙K))	0.1050	0.0992
±Std dev.	(W/(m∙K))	0.0007	0.0009

**Table 5:** Vapor permeability results.

Measured Parameters		Filler/RH Condition			
		THS		FHS	
symbol (unit)	name	0–50%	50–94%	0–50%	50–94%
δ (kg/(m·s·Pa))	water vapor permeability	1.62 × 10^−11^	3.55 × 10^−11^	1.60 × 10^−11^	4.01 × 10^−11^
μ (-)	vapor diffusion resistance factor	12.18	5.57	12.37	4.94

**Table 6:** Specific heat at T = 30 °C.

Temperature of Measurement	Type	Specific Heat (J/(kg·K))				
		Sample 1	Sample 2	Sample 3	X_avg_	Std Dev.
T = 30 °C	FSH	1667	1476	1582	1575	95.7
	TSH	1537	1675	1592	1601	69.5
